# Inspiratory muscle training to reduce risk of pulmonary complications after coronary artery bypass grafting: a systematic review and meta-analysis

**DOI:** 10.3389/fcvm.2023.1223619

**Published:** 2023-07-24

**Authors:** Yuping Xiang, Qin Zhao, Tinahui Luo, Ling Zeng

**Affiliations:** Department of Critical Care Medicine, West China Hospital, Sichuan University/West China School of Nursing, Sichuan University, Chengdu, China

**Keywords:** inspiratory muscle training, postoperative pulmonary complications, coronary artery bypass grafting, meta-analysis, randomized controlled trials

## Abstract

**Background:**

Pulmonary complications occur in a substantial proportion of patients who undergo coronary artery bypass grafting. Inspiratory muscle training (IMT), a simple, well-tolerated physical therapy, has been proposed to reduce the risk of complications, but its efficacy remains controversial.

**Method:**

Randomized controlled trials (RCTs) examining the influence of IMT on the risk of pulmonary complications after coronary artery bypass grafting were identified from PubMed, Embase, CENTRAL, CINAL, and Web of Science through March 2023. Data were meta-analyzed for the primary outcomes of pulmonary complications, defined as pneumonia, pleural effusion, and atelectasis; and in terms of the secondary outcomes of maximum inspiratory pressure, maximum expiratory pressure, length of hospitalization, 6 min walk test, and peak expiratory flow and other outcomes. Risk of bias and quality of evidence assessments were carried out using the RoB 2.0 and Grading of Recommendations Assessment, Development and Evaluation (GRADE) applied to primary outcomes of pulmonary complications.

**Results:**

Data from eight RCTs involving 755 patients were meta-analyzed. IMT was associated with a significantly lower risk of postoperative pneumonia [relative risk (RR) 0.39, 95% confidence interval (CI) 0.25–0.62, *P *< 0.0001] and atelectasis (RR 0.43, 95% CI 0.27–0.67, *P* = 0.0002), but not pleural effusion (RR 1.09, 95% CI 0.62–1.93, *P *= 0.76). IMT was associated with significantly better maximum inspiratory pressure (preoperative: mean difference (MD) 16.55 cmH_2_O, 95% CI 13.86–19.24, *P *< 0.00001; postoperative: mean difference (MD) 8.99 cmH_2_O, 95% CI 2.39–15.60, *P* = 0.008) and maximum expiratory pressure (MD 7.15 cmH_2_O, 95% CI: 1.52–12.79, *P* = 0.01), and with significantly shorter hospitalization (MD −1.71 days, 95% CI −2.56 to −0.87, *P *< 0.001). IMT did not significantly affect peak expiratory flow or distance traveled during the 6 min walk test.

**Conclusions:**

The available evidence from medium and high quality trials suggests that IMT can significantly decrease the risk of pneumonia and atelectasis after coronary artery bypass grafting while shortening hospitalization and improving the strength of respiratory muscles.

**Systematic Review Registration:**

https://www.crd.york.ac.uk/prospero/, identifier: CRD42023415817.

## Introduction

Cardiovascular disease, which is fast becoming the leading cause of death and disability worldwide (1) and caused 17.8 million deaths globally in 2017 (2), is a major health concern together with coronary heart disease, which has a prevalence of 6%–7% in North America, Europe, and Asia (3). Common forms of these diseases are stenosis of the left main coronary artery and multivessel disease, which are typically treated through surgery involving coronary artery bypass grafting (CABG) (4). Such grafting involves substantial risk of pulmonary complications including pneumonia, atelectasis, respiratory failure, pleural effusion, acute respiratory distress syndrome, and pneumothorax (5, 6). These complications can lead to hypoxemia, which affects 11%–40% of patients who undergo CABG (7), and they can prolong hospitalization and increase healthcare costs (8).

Numerous factors appear to contribute to postoperative pulmonary complications after CABG, and a major one is inadequate respiratory muscle function (9). One quarter of patients awaiting elective cardiac surgery show inspiratory muscle weakness (10), which reduces vital capacity, tidal volume, and total lung capacity. It leads to insufficient cough, increasing risk of atelectasis, and pneumonia. Risk of pulmonary complications after CABG can be reduced through respiratory physiotherapy, epidural analgesia, and enhanced recovery protocols (11). Several meta-analyses have concluded that inspiratory muscle training (IMT) can strengthen respiratory muscles and prevent muscle fatigue (12–16). In addition, some scholars have reported that IMT can reduce the incidence of postoperative pulmonary complications (PPCs) (17–19) and short hospitalization (20), but these previous analyses were not comprised of only CABG patients.

A meta-analysis confirmed that IMT had a reduced risk of postoperative pneumonia for CABG patients, which included four studies, and the cutoff time for inclusion was 2017 (21). In recent years, several randomized controlled trials (RCTs) have examined the effects of IMT on PPCs (including pneumonia, atelectasis, and pleural effusion) after CABG. Therefore, we systematically reviewed the literature to identify such RCTs and meta-analyzed their data in order to provide a rigorous assessment of available evidence.

## Methods

This systematic review and meta-analysis were conducted in accordance with the “Preferred Reporting Items for Systematic Reviews and Meta-Analyses” (PRISMA) guidelines and were registered with the identifier CRD42023415817.

### Literature search and study inclusion

We systematically searched the following databases for RCTs indexed through March 2023: PubMed, Embase, Cochrane Library (CENTRAL), CINAL (via EBSCO), and Web of Science. Search strings contained the terms coronary artery bypass [as Medical Subject Heading], coronary artery bypass*, coronary artery bypass surgery, aortocoronary bypass*, coronary artery bypass grafting, CABG, myocardial revascularization, vascular grafting, coronary artery bypass graft; AND breathing exercises [as Medical Subject Heading], breathing exercise*, respiratory muscle training, inspiratory muscle training, expiratory muscle training, inspiratory muscle strength, respiratory exercise, inspiratory muscle train*, respiratory train, ventilatory train, breathing train, respiratory therapy, IMT, RMT (Supplementary Appendix 1). In addition, we manually searched the reference lists of relevant studies.

To be included in this review and meta-analysis, studies had to (1) examine patients at least 18 years old who underwent CABG either pre- or postoperatively; (2) apply a randomized controlled design in which the IMT arm was compared to an arm that received sham IMT, physical therapy, or usual care; (3) report data on at least one of pneumonia, pleural effusion, or atelectasis; and (4) the language of the publication was not limited. Studies were excluded if the full text was unavailable or if they were reviews or observational studies.

### Data extraction and outcomes

Two authors independently extracted the following data using a data extraction form developed *a priori*: (1) study characteristics, including author name, title, comparison arms, and year of publication; (2) population characteristics, including age, sex, and sample size; (3) details of interventions, including training type, frequency, session duration, and intensity; and (4) outcomes. Primary outcomes were rates of pneumonia, pleural effusion, and atelectasis. Secondary outcomes were maximum inspiratory pressure (MIP), maximum expiratory pressure (MEP), Pm_peak_/Pi_max_, and length of hospitalization (LOS). Other outcomes such as exercise capacity were meta-analyzed when relevant data were reported. If outcome data were unclear or not reported, we contacted the authors in an attempt to obtain missing data.

### Assessment of study quality

Two authors independently assessed the risk of bias for included studies using the RoB 2.0 tools for randomized trials and included the following domains: randomization/allocation process, deviation from intended intervention, missing outcome data, outcome measurement, and selective outcome reporting. Visualization of RoB 2.0 was produced using robvis. Studies were judged to be at low, high, or unclear risk of bias. Similarly, discordance was dealt with by adjudication among the authors until consensus was reached by following the appropriate algorithms.

### Statistical analysis

Statistical analyses were conducted using RevMan 5.3 software. Statistical heterogeneity in pooled results was assessed using the chi-squared test, Cochran's Q-test, and the inconsistency *I*^2^ test, in which *I*^2^ values of 25%, 50%, or 75% were considered cut-offs to indicate low, moderate, or high heterogeneity, respectively (22). We performed random-effect heterogeneity in the event of moderate or high heterogeneity. Where appropriate, results were reported as relative risk (RR) and associated 95% confidence interval (CI). The mean difference (MD) was used as the effect size if studies used the same tool to measure the outcome. We used the Grading of Recommendations Assessment, Development and Evaluation (GRADE) system to assess the quality of the body of evidence associated with the following specific outcomes in our review and constructed a summary of findings for the main outcomes: pneumonia, atelectasis, and pleural effusion. This assessment considers the study methodological quality, directness of the evidence, heterogeneity of the data, precision of the effect estimates, and the risk of publication bias.

## Results

### Study selection

**Figure 1:**
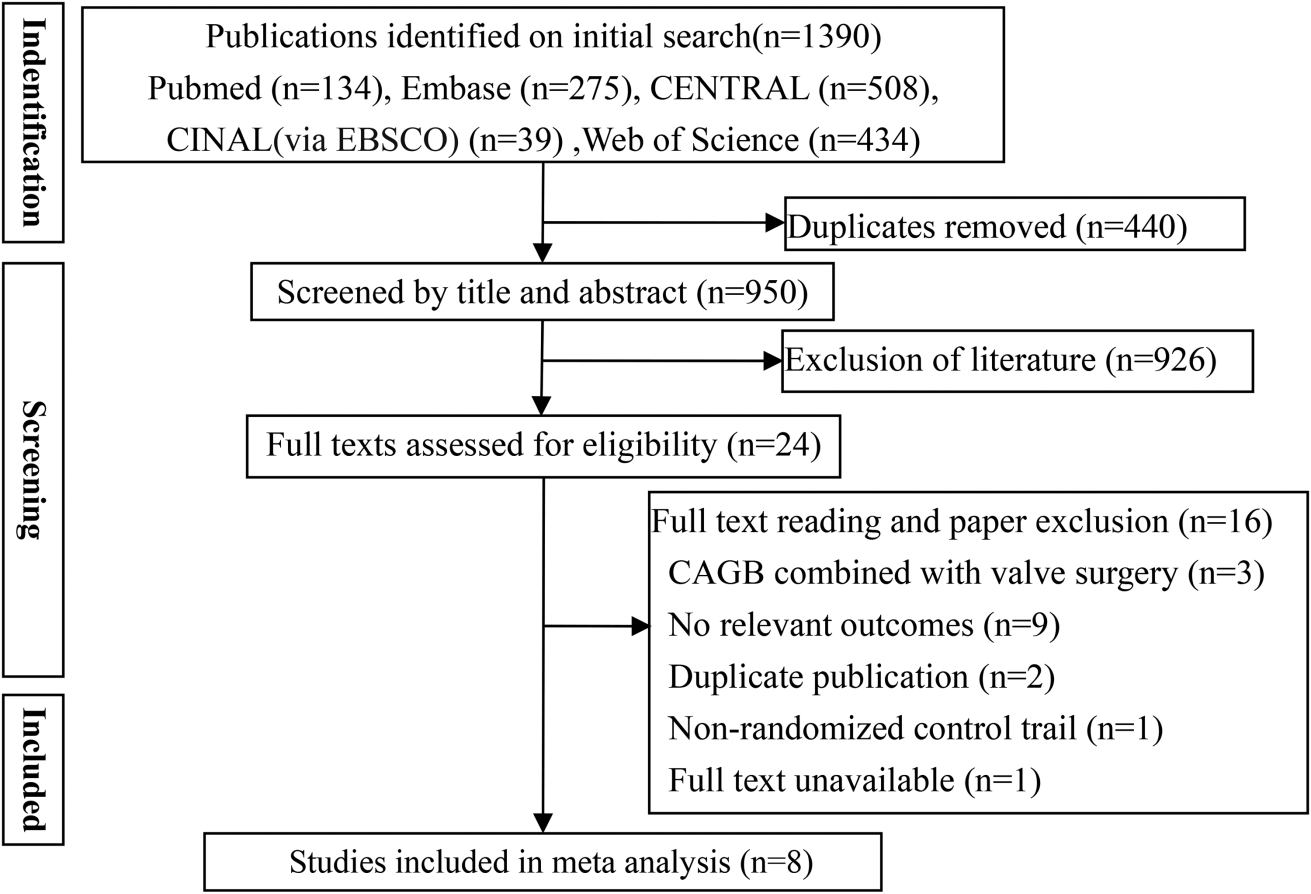
Flowchart of study selection.

Database search yielded 1,390 potentially eligible articles, while manual searching of reference lists did not identify additional studies. After excluding 440 duplicates and 926 studies based on their titles and abstracts, the full text of 24 publications was examined, leading to the exclusion of 16 studies (Figure 1). In the end, a total of eight studies (23–30) met the inclusion criteria and were utilized for meta-analysis (Table 1).

**Table 1:** Characteristics of included studies.

Study	Country	Sample size			Age, years		IMT timing	Type of intervention		Outcomes reported
		*N*	IMT	Ctrl	IMT	Ctrl		IMT	Ctrl	
Weiner et al. (1988) (23)	Israel	84	42	42	59.2 ± 3.8	63.8 ± 3.1	Preop	IMT	Sham IMT	Pneumonia, pleural effusion, FVC, FEV_1_, pH, PaO_2_, PaCO_2_, MIP, Pm_peak_/Pi_max_
Hulzebos et al. (2006) (24)	Netherlands	26	14	12	70.1 ± 9.9	70.5 ± 10.1	Preop	IMT	Usual care	Pneumonia, pleural effusion, segmental atelectasis, MIP, FEV_1_, IVC, FEV_1_/IVC, LOS
Hulzebos et al. (2006) (25)	Netherlands	276	139	137	66.5 ± 9.0	67.3 ± 9.2	Preop	IMT	Usual care	Pneumonia, PPCs, MIP, Pm_peak_/Pi_max_, LOS
Stein et al. (2009) (26)	Brazil	20	10	10	64 ± 7	63 ± 6	Postop	IMT	Usual care	Pneumonia, pleural effusion, atelectasis, 6MWT
Matheus et al. (2012) (27)	Brazil	47	23	24	61.83 ± 13.53	66.33 ± 10.20	Postop	IMT	Physiotherapy	Pneumonia, pleural effusion, atelectasis, MIP, MEP, TV, VC, PEF, LOS
Valkenet et al. (2017) (28)	Netherlands	235	119	116	66 ± 9.2	67.5 ± 9.7	Preop	IMT	Physical therapy	Pneumonia, MIP, LOS, QoL
Cordeiro et al. (2021) (29)	Brazil	38	19	19	55 ± 10	54 ± 10	Postop	IMT	Usual care	Acute lung edema, pneumonia, pleural effusion, atelectasis, reintubation, MIP, MEP, PEF, LOS, 6MWT, FIM
Cordeiro et al. (2022) (30)	Brazil	29	15	14	66 ± 3	68 ± 4	Postop	IMT	Usual care	Pneumonia, pleural effusion, atelectasis, reintubation, pneumothorax, MIP, MEP, PEF

Values are *n* or mean ± SD, unless otherwise specified.

Ctrl, control; PEF, peak expiratory flow; Postop, postoperative; Preop, preoperative; QoL, quality of life; TV, tidal volume; VC, vital capacity.

### Study characteristics

**Figure 2:**
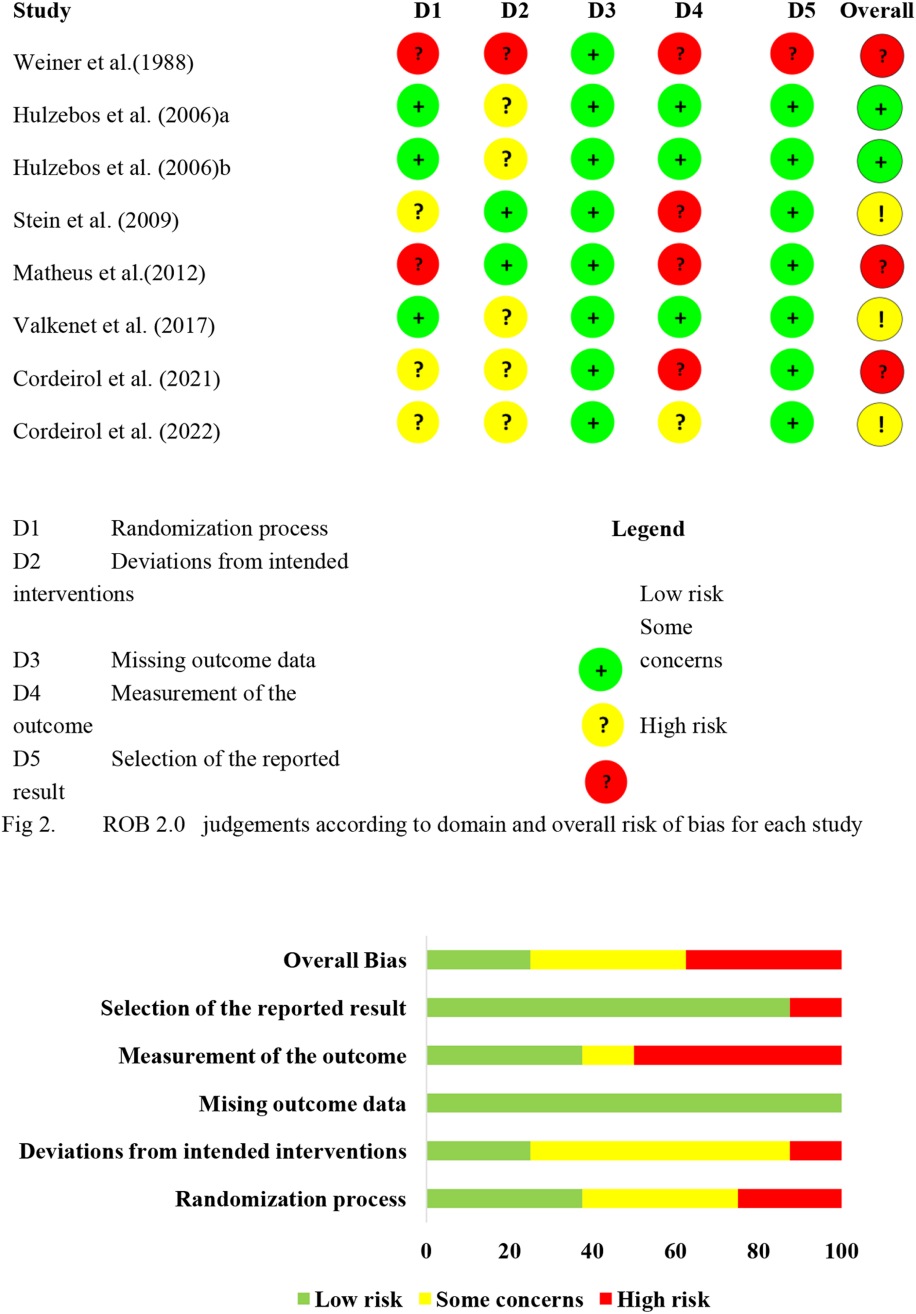
Risk of bias summary. ROB 2.0 judgements according to domain and overall risk of bias for each study.

These studies, each of which enrolled 20–276 patients, involved a total of 755 patients. Studies examined pre- and postoperative IMT, which usually involved the threshold IMT, and involved an intensity at 30%–60% of MIP once to twice daily for durations ranging from 3 days to 4 weeks (Table 2). Most studies described the details of random sequence generation, blinding during outcome assessment, incomplete outcome data, selective reporting, and other biases (Figure 2). However, few studies described details of allocation concealment or blinding of participants and investigators.

**Table 2:** Details of IMT training in included studies.

Study	Equipment	Intensity	Sessions				Total IMT duration
			Duration	No. per day	No. per week	Supervised	
Weiner et al. (1988) (23)	Threshold IMT (Health Scan, NJ, United States)	15% of MIP for 1 week, 5% each session up to 60% of MIP	30 min	1	6	All sessions	2–4 weeks
Hulzebos et al. (2006) (24)	Threshold IMT (PT Medical, Leek, Netherlands)	30% of MIP, RPE <5, the resistance of the inspiratory threshold trainer increased 2 cmH_2_O	20 min	1	7	One session per week	At least 2 weeks
Hulzebos et al. (2006) (25)	Threshold IMT, inspiratory threshold-loading device	30% of MIP, RPE <5, resistance of the inspiratory threshold trainer was increased by 5%	20 min	1	7	One session per week	At least 2 weeks
Stein et al. (2009) (26)	Expiratory positive airway pressure mask	12–18 breaths per min during mask use, expiratory pressure increased by 5–8 cmH_2_O	5–8 min	1	7	NR	6 days
Matheus et al. (2012) (27)	IMT Respironics® Threshold®	40% of the MIP; rhythm and pauses were determined for each patient	Three sets of 10 repetitions	2	NR	NR	3 days
Valkenet et al. (2017) (28)	Threshold IMT, Respironics New Jersey Inc., Cedar Grove, NJ, United States)	30% of MIP, RPE <5, inspiratory load was increased in steps of 5% of the threshold	20 min	1	7	One session per week	At least 2 weeks
Cordeiro et al. (2021) (29)	Linear pressure load device (Threshold IMT®)	40% of MIP	Three sets of 10 repetitions	2	NR	NR	Until discharge
Cordeiro et al. (2022) (30)	Linear pressure loading device (PowerBreathe Kinetic Series, HaB International, United Kingdom)	40% of MIP	Three sets of 15 repetitions	2	NR	Physical therapist	Until discharge

NR, not reported; RPE, rate of perceived exertion.

### Methodological quality

Figure 2 shows the results of the risk of bias assessment.

### Postoperative pulmonary complications

**Figure 3:**
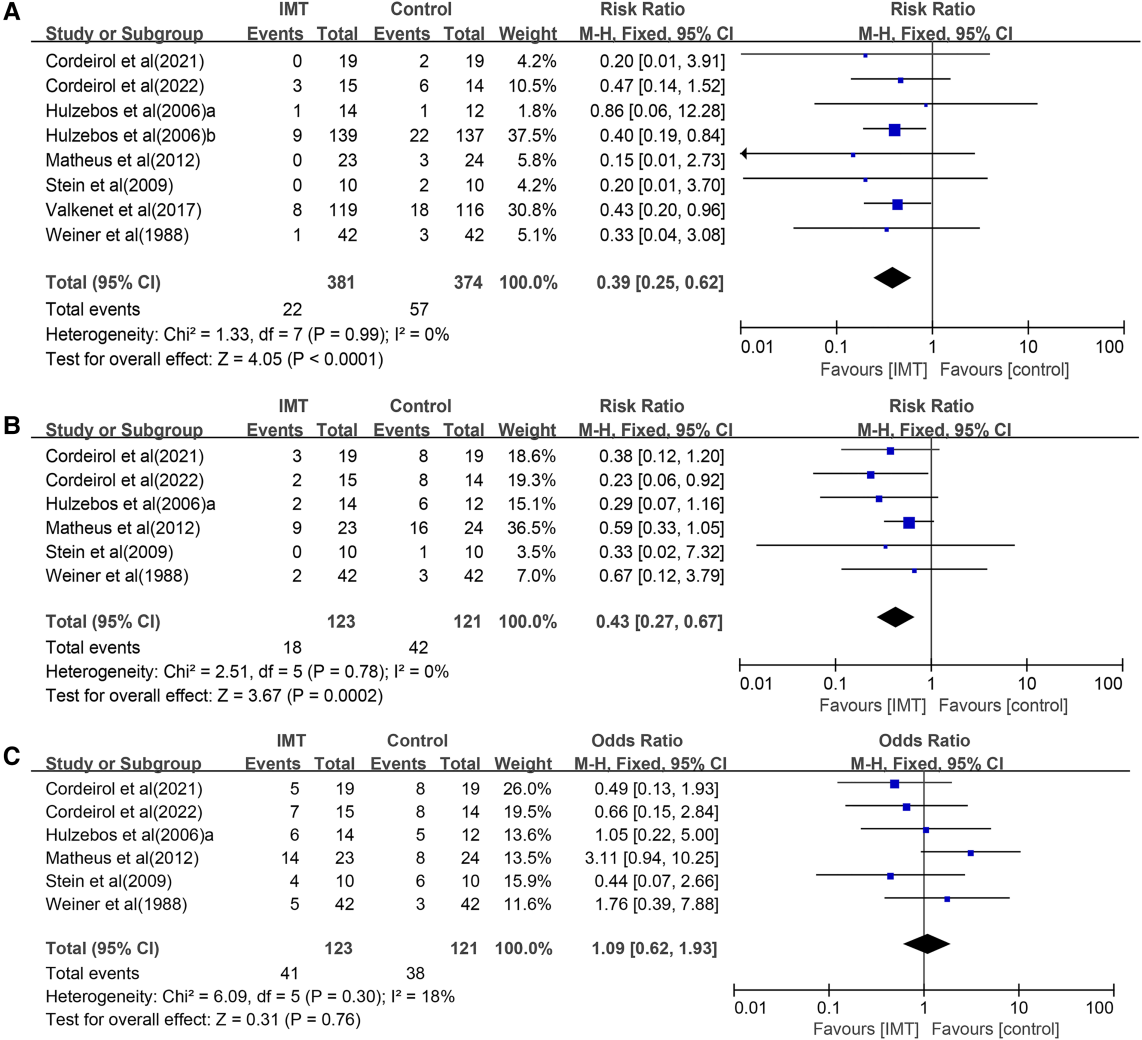
Forest plots of the association between IMT and risk of the postoperative pulmonary complications: (**A**) pneumonia, (**B**) atelectasis, and (**C**) pleural effusion.

(1) All eight studies (23–30) involving 755 participants reported on postoperative pneumonia, the risk of which was significantly lower in the IMT group (RR 0.39, 95% CI 0.25–0.62, *P *< 0.0001; Figure 3A). Although we noted no heterogeneity in the included studies, small-study effects may be present. We downgraded the outcome for risk of bias and publication bias, but upgraded it for large effect (RR < 0.5). As a result, we rated the quality of evidence as moderate quality.
(2) Six trials (23, 24, 26, 27, 29, 30) involving 244 participants reported on postoperative atelectasis, the risk of which was significantly lower in the IMT group (RR 0.43, 95% CI 0.27–0.67, *P *= 0.0002; Figure 3B). Although we noted no heterogeneity in the included studies, small-study effects may be present. We therefore downgraded the outcome for risk of bias and publication bias. As a result, we rated the quality of evidence as low quality.
(3) Six trials (23, 24, 26, 27, 29, 30) reported on postoperative pleural effusion, the risk of which was not significantly different between the IMT and control groups (RR 1.09, 95% CI 0.62–1.93, *P *= 0.76; Figure 3C). The three outcomes were meta-analyzed using a fixed-effect model because of low heterogeneity.

We noted a low heterogeneity between the trials, and small-study effects may be present. So we downgraded this outcome for inconsistency and publication bias. Eventually, we rated the quality of evidence as very low quality and the effect is uncertain.

### Strength and endurance of respiratory muscles

**Figure 4:**
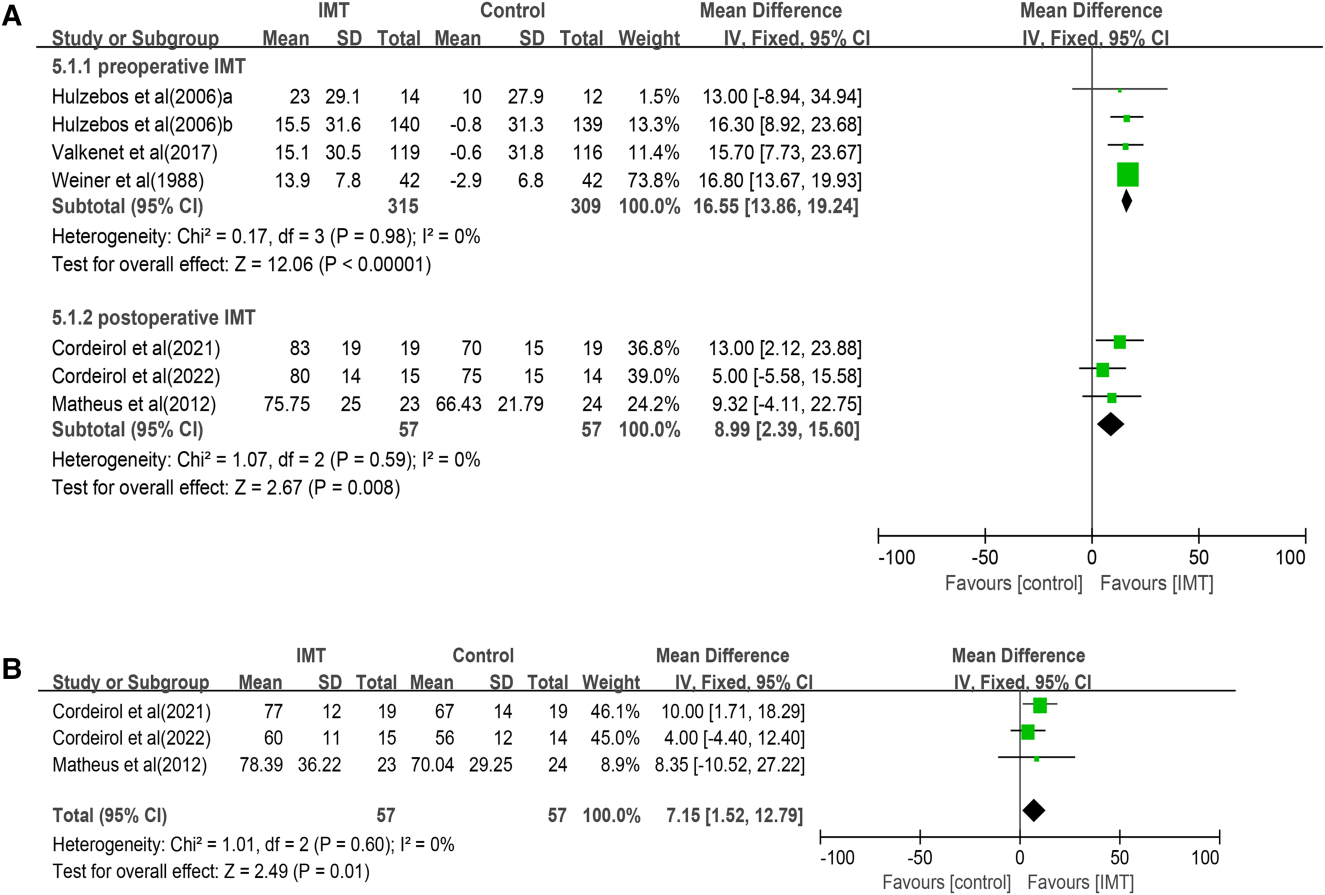
Forrest plot of the effect of IMT on respiratory muscle strength: (**A**) the change of MIP and (**B**) the change of MEP.

Seven studies involving 738 participants evaluated the effect of IMT on MIP (23–25, 27–30), and it was significantly better in the IMT group. Similar results were obtained in separate meta-analyses of the four studies involving preoperative IMT (MD 16.55 cmH_2_O, 95% CI 13.86–19.24, *P *< 0.00001); or the remaining three studies involving postoperative IMT (MD 8.99 cmH_2_O, 95% CI: 2.39–15.60, *P* = 0.008) (Figure 4A).

Meta-analysis of three studies (27, 29, 30) involving 144 patients associated postoperative IMT with significantly better MEP (MD 7.15 cmH_2_O, 95% CI 1.52–12.79, *P* = 0.01; Figure 4B). Both meta-analyses were performed with fixed-effect models because heterogeneity was negligible (*I*^2^ = 0%). Two trials (23, 25) reported the data about respiratory muscle endurance, which they assessed in terms of the ratio of Pm_peak_ to Pi_max_ (Table 3), but data heterogeneity were too high to permit meta-analysis (*I*^2 ^= 90%, *P *= 0.018).

**Table 3:** Summary of outcomes in included studies^a^.

Study	Postoperative pulmonary complications	Respiratory muscle strength	Other outcomes		
Weiner et al. (1988) (23)	Pneumonia: 1 vs. 3 Pleural effusion: 5 vs. 3 Hemidiaphragmatic paralysis: 2 vs. 3	MIP: 101.9 ± 7.8 vs. 91.2 ± 6.8	Inspiratory muscle endurance Pm_peak_/Pi_max_(%): 87.0 ± 5.2 vs. 75.6 ± 4.8		
Hulzebos et al. (2006) (24)	Pneumonia: 1 vs. 1 Pleural effusion: 6 vs. 5 Segmental atelectasis: 2 vs. 6	MIP: 87.6 ± 29.1 vs. 76.8 ± 27.9	LOS: 7.93 ± 1.94 vs. 9.92 ± 5.78		Lung function FEV_1_: 80.7 ± 20.6 vs. 80.9 ± 20.3 IVC%: 87.3 ± 18.1 vs. 87.4 ± 17.8 FEV_1_/IVC: 93.0 ± 9.6 vs. 94.7 ± 17.6
Hulzebos et al. (2006) (25)	Pneumonia: 9 vs. 22 Total postoperative pulmonary complications: 25 vs. 48	MIP: 95.6 ± 31.6 vs. 79.5 ± 31.3	LOS: 7 (5–41) vs. 8 (6–70)		Inspiratory muscle endurance Pm_peak_/Pi_max_(%):56.0 ± 15.1 vs. 51.8 ± 16.4
Stein et al. (2009) (26)	Pneumonia: 0 vs. 2 Pleural effusion: 4 vs. 6 Atelectasis: 0 vs. 1		Functional capacity 6 min walk test: 416 ± 78 vs. 323 ± 67		
Matheus et al. (2012) (27)	Pneumonia: 0 vs. 3 Pleural effusion: 14 vs. 8 atelectasis: 9 vs. 16	MIP:75.75 ± 25.00 vs. 66.43 ± 21.79 MEP: 78.39 ± 36.22 vs. 70.04 ± 29.25	LOS: 6.2 ± 2.02 vs. 6.77 ± 2.95		Lung function ① TV: *P* = 0.0,490 ② VC: 1,230.4 ± 477.86 vs. 919.17 ± 394.47 ③ PEF: 221.30 ± 100.87 vs. 203.75 ± 83.55
Valkenet et al. (2017) (28)	Pneumonia: 8 vs. 18	MIP: 99.0 ± 30.5 vs. 81.2 ± 31.8	LOS: 8.2 ± 2.6 vs. 10.0 ± 7.8		QoL: there were no significant differences in change of QoL scores
Cordeiro et al. (2021) (29)	Acute lung edema: 5 vs. 8 Pneumonia: 0 vs. 2 Pleural effusion: 5 vs. 8 Atelectasis: 3 vs. 8 Reintubation: 1 vs. 2	MIP: 83 ± 19 vs. 70 ± 15 MEP: 77 ± 12 vs. 67 ± 14	LOS: 6 ± 2 vs. 9 ± 3	Lung function PEF: 311 ± 15 vs. 231 ± 21	functional capacity 6MWT: 398 ± 20 vs. 305 ± 21 Functionality assessment FIM: 120 ± 3 vs. 112 ± 5
Cordeiro et al. (2022) (30)	Pneumonia:3 vs. 6 Pleural effusion:7 vs.8 Atelectasis: 2 vs. 8 Reintubation: 3 vs. 6 Pneumothorax: 1 vs. 1	MIP: 80 ± 14 vs. 75 ± 15 MEP:60 ± 11 vs. 56 ± 12	Functional capacity 6 min walk test: 285 ± 51 vs. 288 ± 45		

^a^ Values are *n* or mean ± SD for the IMT group vs. the control group unless otherwise specified.

### Length of hospitalization

**Figure 5:**
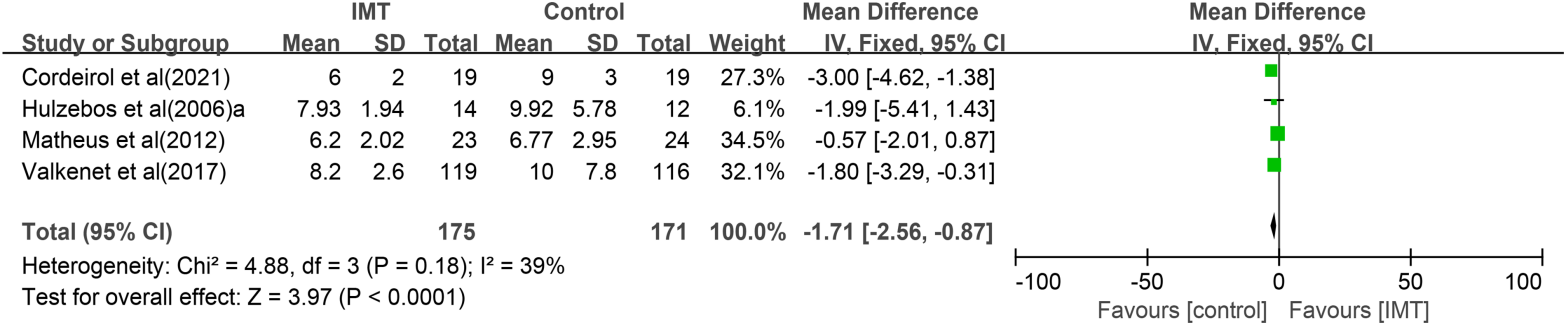
Forest plot of the association between IMT and length of hospitalization.

Five studies (24, 25, 27–29) compared length of hospitalization between the IMT and control groups, but one (25) of them reported respective median and ranges [7 (5–41) vs. 8 (6–70) days], which we could not pool with the means and standard deviations reported in the other four trials. Meta-analysis of those four trials associated IMT with significantly shorter hospitalization (−1.71 days, 95% CI −2.56 to −0.87 days, *P *< 0.0001; Figure 5).

### Exercise capacity

**Figure 6:**
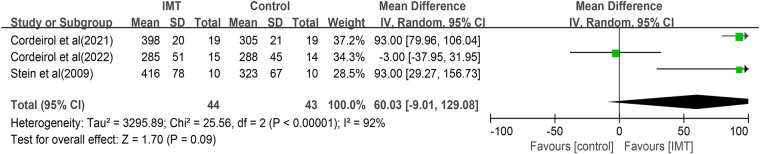
Forest plot of the association between IMT and distance traveled in the 6 min walk test.

Three studies (26, 29, 30) examined the impact of IMT on performance in the 6 min walk test (6MWT). Due to the high heterogeneity (*I*^2 ^= 92%, *P *< 0.00001), a random effects model was used for pooled analysis. The results showed that IMT was not associated with significantly longer distance traveled (MD 60.03 m, 95% CI −9.01 to 129.08, *P *= 0.09; Figure 6). Due to the small sample size, subgroup analysis and meta-regression cannot be conducted.

### Other outcomes

**Figure 7:**
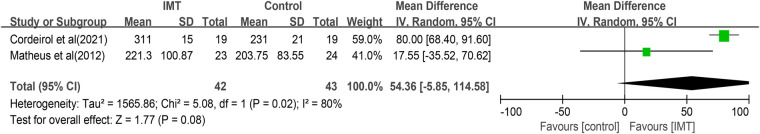
Forest plot of the association between IMT and peak expiratory flow.

Subsets of the eight RCTs reported on additional secondary outcomes. One trial (28) with 235 participants found no significant difference in postoperative quality of life between IMT and control groups, as measured using the Form 36 questionnaire or the EuroQol questionnaire with five dimensions and three levels. One trial (28) reported no significant difference between IMT and control groups in how much percentage of inspiratory vital capacity (IVC%), forced vital capacity (FVC%), and forced expiration volume in 1 s (FEV_1_%) changed as a result of CABG (Table 3). Similarly, another study (27) reported improvement between IMT and control groups in tidal volume (*P *= 0.049) and vital capacity (*P *= 0.022). Meta-analysis of two studies (27, 29) involving 85 patients found that IMT did not significantly affect the peak expiratory flow (MD 54.36 L/min, 95% CI −5.85 to 114.58, *P* = 0.08; Figure 7).

## Discussion

This review of high-quality RCTs suggests that IMT can significantly decrease the risk of pulmonary complications like pneumonia and atelectasis after CABG, although not necessarily a risk of pleural effusion. In addition, IMT can significantly shorten hospitalization and strengthen respiratory muscles. Conclusions should be interpreted with caution in light of the apparent risk of overestimating the benefits of IMT, given the lack of adequate blinding, small samples, and publication bias in the available evidence.

Previous systematic reviews did show evidence that IMT can reduce PPCs in patients undergoing cardiac surgery. The meta-analysis by Ge et al. (19) showed that preoperative IMT resulted in significantly improved MIP and was associated with decreased PPCs, for patients undergoing cardiac, upper abdominal, and thoracic surgery. Gomes Neto et al. (13) showed that preoperative IMT reduced the risk of PPCs in patients undergoing cardiac surgery, which included three trials with 386 patients, and improved the MIP and reduced the length of hospital stay. A Cochrane meta-analysis (18) showed that preoperative IMT was associated with a reduction of postoperative atelectasis, pneumonia, and length of hospital stay in adults undergoing cardiac and major abdominal surgery, which included 12 trials with 695 participants. In addition, Thybo Karanfil and Møller (17) confirmed that preoperative IMT may reduce the risk of pneumonia and atelectasis after cardiac surgery, which included five trials with 348 patients. Thus, above-mentioned studies have focused on patients after cardiac surgery, and the definition of PPCs is inconsistent. Recently, the meta-analysis by Zhang et al. (21) revealed that IMT can improve the inspiratory muscle strength and endurance, pulmonary function, and 6MWT, and decrease PPCs and the LOS, which included 12 trials with 918 patients. However, only four studies that reported postoperative pneumonia were pooled analysis, and the cutoff time for inclusion was 2017, which is in accordance with our findings.

### Limitations

The limitations of our systematic review and meta-analysis should also be noted. First, although the overall analysis suggests that IMT can reduce pneumonia and atelectasis, further studies are needed to examine IMT on different types of PPCs, including pleural effusion, mechanical ventilation more than 48 h, and pneumothorax. Additionally, preoperative and postoperative intervention period were included, and subgroup analysis was not performed, except MIP. Second, in the secondary outcome, the quality of life and pulmonary function (FVC, FEV_1_) data cannot be completely extracted. Thus, meta-analysis was not done. Third, this review did not cover cost–benefit and safety analyses. Fourth, due to the intervention equipment, sessions and intensity were inconsistent, and subgroup analysis was not performed. More well-designed large research studies are still needed.

## Data Availability

The raw data supporting the conclusions of this article will be made available by the authors, without undue reservation.
