# Supplementary material for: Inspiratory muscle training to reduce risk of pulmonary complications after coronary artery bypass grafting: a systematic review and meta-analysis
Source: Front Cardiovasc Med. 2023 Jul 24;10:1223619. doi: 10.3389/fcvm.2023.1223619 (PMC10408668; doi:10.3389/fcvm.2023.1223619)
Supplement: Supplementary file 1 [file Table1.docx]

Supplementary Material

**Inspiratory muscle training to reduce risk of pulmonary complications after coronary artery bypass grafting: a systematic review and meta-analysis**

Yuping Xiang, Qin Zhao, Tinahui Luo, Ling Zeng*

*** Correspondence:** Ling zeng. Email: zengling510@163.com

1.Supplementary Literature search

**1.Pubmed（134）**

#1 "Breathing Exercises"[Mesh]  （4162）

#2 ((((((((((((breathing Exercise*[Title/Abstract]) OR (respiratory muscle training[Title/Abstract])) OR (inspiratory muscle training[Title/Abstract])) OR (expiratory muscle training[Title/Abstract])) OR (respiratory exercise[Title/Abstract])) OR (inspiratory muscle train*[Title/Abstract])) OR (respiratory train[Title/Abstract])) OR (ventilatory train[Title/Abstract])) OR (breathing train[Title/Abstract])) OR (respiratory therapy[Title/Abstract])) OR (IMT[Title/Abstract])) OR (RMT[Title/Abstract])) OR (Inspiratory Muscle Strength[Title/Abstract]) （16196）

#3 #1 OR #2 （18911）

#4 "Coronary Artery Bypass"[Mesh] （56439）

#5 ((((((coronary Artery Bypass*[Title/Abstract]) OR (coronary Artery Bypass Surgery[Title/Abstract])) OR (aortocoronary Bypass*[Title/Abstract])) OR (coronary Artery Bypass Grafting[Title/Abstract])) OR (CABG[Title/Abstract])) OR (myocardial revascularization[Title/Abstract])) OR (vascular Grafting[Title/Abstract])

（55056）

#6 #4 OR #5 （76834）

#7 #3 AND #6 （134）

**2.Embase（275）**

#1 'breathing exercise'/exp（[10,410](https://www.embase.com/)）

#2 'inspiratory muscle training'/exp（[195](https://www.embase.com/)）

#3 'respiratory muscle training'/exp（[51](https://www.embase.com/)）

#4 'inspiratory muscle strength'/exp（[22](https://www.embase.com/)）

#5 'breathing exercise*':ti,ab,kw（[2,664](https://www.embase.com/)）

#6 'expiratory muscle training':ti,ab,kw（[97](https://www.embase.com/)）

#7 'respiratory exercise':ti,ab,kw（[163](https://www.embase.com/)）

#8 'inspiratory muscle train*':ti,ab,kw（[1,409](https://www.embase.com/)）

#9 'respiratory train':ti,ab,kw（[1](https://www.embase.com/)）

#10 'ventilatory train':ti,ab,kw（[0](https://www.embase.com/)）

#11 'breathing train':ti,ab,kw（0）

#12 'respiratory therapy':ti,ab,kw（[2,567](https://www.embase.com/)）

#13 imt:ti,ab,kw（[16,017](https://www.embase.com/)）

#14 rmt:ti,ab,kw（[2,458](https://www.embase.com/)）

#15 #1 OR #2 OR #3 OR #4 OR #5 OR #6 OR #7 OR #8 OR #9 OR #10 OR #11 OR #12 OR #13 OR #14（[32,235](https://www.embase.com/)）

#16 'coronary artery bypass graft'/exp（[85,594](https://www.embase.com/)）

#17 'coronary artery bypass':ti,ab,kw（[63,370](https://www.embase.com/)）

#18 'coronary artery bypass*':ti,ab,kw（[63,447](https://www.embase.com/)）

#19 'coronary artery bypass surgery':ti,ab,kw（[11,602](https://www.embase.com/)）

#20 'aortocoronary bypass*':ti,ab,kw（[2,960](https://www.embase.com/)）

#21 'coronary artery bypass grafting':ti,ab,kw（[34,372](https://www.embase.com/)）

#22 cabg:ti,ab,kw（[38,242](https://www.embase.com/)）

#23 'myocardial revascularization':ti,ab,kw（[6,891](https://www.embase.com/)）

#24 'vascular grafting':ti,ab,kw（[331](https://www.embase.com/)）

#25 #16 OR #17 OR #18 OR #19 OR #20 OR #21 OR #22 OR #23 OR #24（[118,456](https://www.embase.com/)）

#26 #15 AND #25（275）

**3.CINAL (via EBSCO)（39）**

S1 AB breathing exercises OR AB breathing Exercise* OR AB respiratory muscle training OR AB inspiratory muscle training OR AB expiratory muscle training OR AB Inspiratory Muscle Strength OR AB respiratory exercise OR AB inspiratory muscle train* OR AB imt OR AB rmt OR AB breathing train OR AB respiratory therapy （2882）

S2 AB coronary artery bypass OR AB coronary Artery Bypass* OR AB coronary Artery Bypass Surgery OR AB aortocoronary Bypass* OR AB coronary Artery Bypass Grafting OR AB CABG OR AB remyocardial revascularization OR AB vascular Grafting OR AB coronary artery bypass graft （4176）

S3 S1 AND S2 （39）

**4.CENTRAL（508）**

#1 MeSH descriptor: [Coronary Artery Bypass] explode all trees（6216）

#2 (myocardial revascularization):ti,ab,kw OR (vascular Grafting):ti,ab,kw OR (coronary artery bypass graft):ti,ab,kw（19554）

#3 (coronary Artery Bypass Surgery):ti,ab,kw OR (coronary Artery Bypass*):ti,ab,kw OR (aortocoronary Bypass*):ti,ab,kw OR (coronary Artery Bypass Grafting):ti,ab,kw OR (CABG):ti,ab,kw（15062）

#4 #1 OR #2 OR #3（23719）

#5 (breathing exercises):ti,ab,kw OR (breathing Exercise*):ti,ab,kw OR (respiratory muscle training):ti,ab,kw OR (inspiratory muscle training):ti,ab,kw OR (expiratory muscle training):ti,ab,kw（9860）

#6 (respiratory exercise):ti,ab,kw OR (inspiratory muscle train*):ti,ab,kw OR (respiratory train):ti,ab,kw OR (respiratory therapy):ti,ab,kw OR (IMT):ti,ab,kw（46874）

#7 #5 OR #6（51395）

#8 #4 AND #7（508）

**5.Web of science（434）**

#1. (((((((((((((TS=(breathing exercises)) OR TS=(breathing Exercise*)) OR TS=(respiratory muscle training)) OR TS=(inspiratory muscle training)) OR TS=(expiratory muscle training)) OR TS=(Inspiratory Muscle Strength)) OR TS=(respiratory exercise)) OR TS=(inspiratory muscle train*)) OR TS=(respiratory train)) OR TS=(ventilatory train)) OR TS=(breathing train)) OR TS=(respiratory therapy)) OR TS=(IMT)) OR TS=(RMT)（109939）

#2. ((((((((TS=(coronary Artery Bypass)) OR TS=(coronary Artery Bypass*)) OR TS=(coronary Artery Bypass Surgery)) OR TS=(aortocoronary Bypass*)) OR TS=(coronary Artery Bypass Grafting)) OR TS=(CABG)) OR TS=(myocardial revascularization)) OR TS=(vascular Grafting)) OR TS=(coronary artery bypass graft)（97140）

3. #1 AND #2 （434）
