# Supplementary figures and images for: Inspiratory muscle training to reduce risk of pulmonary complications after coronary artery bypass grafting: a systematic review and meta-analysis
Source: Front Cardiovasc Med. 2023 Jul 24;10:1223619. doi: 10.3389/fcvm.2023.1223619 (PMC10408668; doi:10.3389/fcvm.2023.1223619)

Supplementary file 3: Patients characteristics based on interventions.


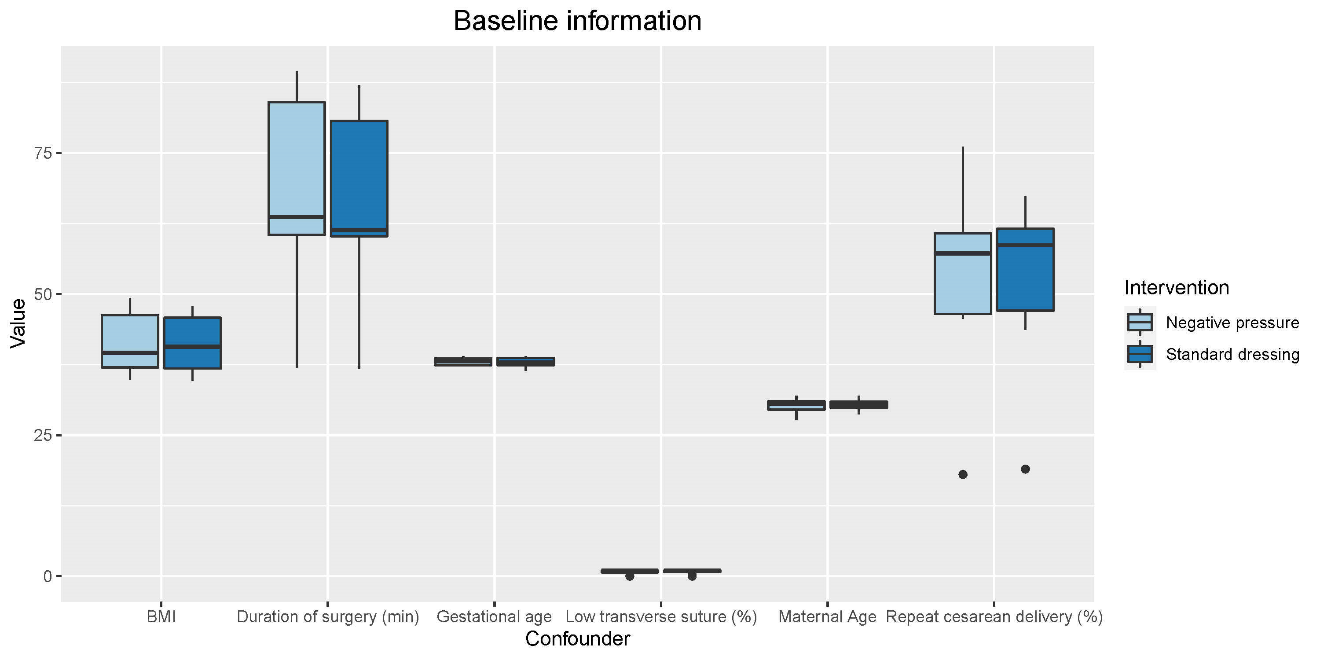

Supplement: Supplementary file 2 [file Datasheet1.docx]
